# Challenges for Predictive Modeling With Neural Network Techniques Using Error‐Prone Dietary Intake Data

**DOI:** 10.1002/sim.70013

**Published:** 2025-02-08

**Authors:** Dylan Spicker, Amir Nazemi, Joy Hutchinson, Paul Fieguth, Sharon Kirkpatrick, Michael Wallace, Kevin W. Dodd

**Affiliations:** ^1^ Department of Mathematics and Statistics University of New Brunswick (Saint John) Saint John New Brunswick Canada; ^2^ Department of Systems Design Engineering University of Waterloo Waterloo Ontario Canada; ^3^ School of Public Health Sciences University of Waterloo Waterloo Ontario Canada; ^4^ Department of Statistics and Actuarial Science University of Waterloo Waterloo Ontario Canada; ^5^ Biometry Research Group, Division of Cancer Prevention National Cancer Institute Bethesda Maryland USA

**Keywords:** artificial neural networks, dietary assessment, dietary data, machine learning, measurement error, prediction

## Abstract

Dietary intake data are routinely drawn upon to explore diet‐health relationships, and inform clinical practice and public health. However, these data are almost always subject to measurement error, distorting true diet‐health relationships. Beyond measurement error, there are likely complex synergistic and sometimes antagonistic interactions between different dietary components, complicating the relationships between diet and health outcomes. Flexible models are required to capture the nuance that these complex interactions introduce. This complexity makes research on diet‐health relationships an appealing candidate for the application of modern machine learning techniques, and in particular, neural networks. Neural networks are computational models that can capture highly complex, nonlinear relationships, so long as sufficient data are available. While these models have been applied in many domains, the impacts of measurement error on the performance of predictive modeling have not been widely investigated. In this work, we demonstrate the ways in which measurement error erodes the performance of neural networks and illustrate the care that is required for leveraging these models in the presence of error. We demonstrate the role that sample size and replicate measurements play in model performance, indicate a motivation for the investigation of transformations to additivity, and illustrate the caution required to prevent model overfitting. While the past performance of neural networks across various domains makes them an attractive candidate for examining diet‐health relationships, our work demonstrates that substantial care and further methodological development are both required to observe increased predictive performance when applying these techniques compared to more traditional statistical procedures.

## 1 Introduction

Measurement error describes scenarios where the observed value of a variate differs from the value we wish to observe. While measurement error is pervasive across many disciplines, it is of particular concern in the analysis of dietary intake data, where reported dietary intake will often differ from true dietary intake [1, 2]. Dietary intake data are typically collected from one of two instruments: food‐frequency questionnaires (FFQs) or 24‐hour recalls (24HRs). FFQs are questionnaires that broadly solicit responses regarding the frequency with which particular foods are consumed, with typical recall periods ranging from a month to a year [1, 3]. In contrast, 24HRs ask individuals to recall what foods were consumed during a specific 24‐hour period [4]. The values measured from instruments directly are referred to as *reported intake*, and both 24HRs and FFQs produce reported intake data that is subject to measurement error [5]. Typically, 24HRs have less systematic error than FFQs; however, a 24HR merely captures a snapshot in time [6, 7]. As a result, repeated measurements of 24HRs are often taken in order to estimate *usual intake* (i.e., the long‐term average intake) of a dietary component. When considering the impact of dietary intake on health outcomes, we most often want to understand the impact of usual intake rather than reported intake. The effects of measurement error on estimation and inference have been thoroughly studied, and the impacts of ignoring measurement error are well understood for many commonly applied statistical estimation techniques [2].

In certain settings, the primary focus of an analysis is on prediction rather than estimation directly. For instance, it is of interest to develop predictive models for the development of breast cancer [8], to be able to preemptively detect seizures in patients with epilepsy [9], to use dietary information to predict coronary heart disease [10], or to predict verbal intelligence during adulthood from adolescent physical activity and nutrition [11]. In these analyses, the primary aim is developing a model with predictive utility rather than explanatory power. As a result, the common concerns of consistency and bias are subordinated to the predictive performance of the models.

The measurement error literature tends to focus on reducing bias or restoring consistency in estimation and on correcting uncertainty quantification for inference, with comparatively less attention paid to problems of prediction. Part of the reason for this is well summarized in an argument put forward by Carroll et al. (Section 2.6) [12]. The authors suggest measurement error is of limited concern in prediction problems since the prediction problem can be reframed to use the error‐prone versions as predictors directly. Consequently, it is statistically convenient to model the outcome of interest from the observed quantities, even if those happen to be error‐prone. In the context of dietary intake, this may mean predicting an individual's systolic blood pressure based not on their usual sodium intake but rather their reported sodium intake.

For the use of observed quantities to be an effective strategy, the analyst must be able to specify a useful predictive model linking the outcome and the error‐prone surrogate measurements. This can be nontrivial since it will generally be the case that a naive substitution of the surrogate measurements into the standard outcome model will result in poor performance. For instance, if linear regression is thought to be suitable to predict the outcome from the true, error‐free variate, there is no guarantee that a linear regression is appropriate when using the surrogate measurement. There may also be substantial loss of predictive performance when building models using error‐prone surrogates compared to the true, error‐free variates. In the context of risk prediction, Khudyakov et al. [13] demonstrate that the use of error‐prone variates in place of the true variate can reduce the performance of predictive models substantially. This reduction in performance may be of particular concern if these limitations are not properly understood, and so the authors recommend consideration when designing studies where there is the possibility of taking more costly measurements that are subject to less error on fewer individuals.

In dietary assessment research, depending on what dietary components are of primary interest, it is often the case that there is no practical way to obtain even an asymptotically error‐free measure of true intake. One technique is to directly observe individuals over time; however, this is not feasible over long periods of time or with large samples. Beyond observation, there are four available *recovery biomarkers*, assessment instruments that are subject exclusively to random noise and thus can be used to approximate true intake via averaging. These are: doubly labelled water to assess total energy intake, 24‐hour urinary sodium excretion to assess sodium intake, 24‐hour urinary potassium excretion to assess potassium intake, and 24‐hour urinary nitrogen excretion to assess protein intake [14]. These biomarkers assess total intake of the specific components they measure; however, they cannot assess the total diet and are both burdensome and expensive to administer. Thus, researchers generally rely on self‐report assessment instruments that can capture total dietary intake but are prone to both bias and error. When using 24HRs, researchers may take several repeated measurements across different days to better estimate usual intake. While this procedure can partially ameliorate concerns relating to error and bias in usual intake estimation, increasing the number of observations per individual results in high participant burden, and the quality of responses decreases as the number of responses increases [15]. Of final note, in order for the argument regarding ignoring errors in predictive modeling to hold, it must be the case that the measurement error models are *transportable*. If the data from one population are used to construct a predictive model that is to be applied in a different population, then the modeling strategy based on surrogate measurements is only valid when the errors in each population are drawn from the same distribution [12, 16, 17].

While the loss of predictive performance and the requirement of transportability are innate features of the statistical structures under consideration, the need for correct model specification can be addressed through the careful selection of modeling strategies. An analyst may, for instance, choose nonparametric techniques that can capture the underlying relationship effectively, regardless of the true complexity of that relationship. In light of modern advances in computer science, one strategy that has demonstrated particular efficacy in complex modeling scenarios is the use of *artificial neural networks*. Artificial neural networks (hereafter referred to as simply neural networks) are computational models, loosely designed to mimic the structure of neurons in a brain, that are commonly deployed for the purpose of machine learning [18, 19]. From a statistical perspective, neural networks can be viewed as either a semiparametric or nonparametric technique, where sufficient parameters can be introduced to capture highly complex relationships without the need for direct specification. As a result of advances in the computational feasibility of *deep neural networks* (that is, neural networks with very large numbers of parameters) and with growing access to data, neural networks have been successfully employed in many complex prediction settings. For instance, neural networks have been used to predict crop yields from weather data [20], reaction types in organic chemistry [21], user responses in online advertising contexts [22], diet quality through a healthy eating index score [23], and clinical events using electronic health record data [24]. Neural networks are also able to incorporate diverse types of data, both as explanatory variates and outcomes, and are able to make effective use of very large data. The promise of using neural networks, and machine learning more broadly, to allow for flexible, complex, predictive models has generated interest in the context of modeling diet‐health relationships.

A range of techniques has been applied, demonstrating the potential utility of machine learning algorithms for understanding diet‐health relationships. To date, there has been less of an effort to ensure that these approaches are being applied appropriately with dietary intake data. While the consideration of measurement error for traditional statistical modeling for dietary intake data has received a great deal of attention, applications of machine learning to dietary intake data have typically not considered the effects of measurement error. This has been the case both for models that are predominantly concerned with prediction and for models that admit easier interpretations. In the machine learning literature, separate from nutrition research, concerns relating to measurement error have also been left mostly unaddressed. A notable exception is the work of Hu et al. [25] These authors investigate the use of neural networks to fit predictive models in the presence of measurement error while simultaneously learning the error‐free distribution of the predictors. They use a *normalizing flow* to approximate the error‐free distribution and fit a network simultaneously to estimate a regression function. This is a promising line of research that indicates how measurement error may be accommodated in neural networks. The techniques require parametric assumptions on the error distribution, and assume that the parameters of this distribution are known. This makes the technique not directly suited to the context of dietary intake data; however, extensions to this work provide a possible avenue for future work.

Despite the nascent theory for addressing measurement error, machine learning models have been investigated for dietary intake analysis. Morgenstern et al. [26] use conditional inference forest models to relate reported nutrient intake on a given day to cardiovascular disease risk, building predictive models. Panaretos et al. [27] show that machine learning methods have better predictive performance compared with linear regression in the classification of long‐term cardiometabolic risk when using individuals' dietary patterns. Bodnar et al. [28] use the Super Learner algorithm to investigate the relationship between fruit and vegetable intake and adverse outcomes in pregnancy and contrast these results with a more traditional multivariable logistic regression. Madeira et al. [29] use latent class transition models to assess the association between a posteriori‐derived dietary patterns and nutritional status in older adults. Similarly, Farmer et al. [30] use latent class analysis to explore dietary patterns emerging in US adults. Shang et al. [31] use regression models, random forests, and gradient boosting machines to identify dietary determinants for changes in cardiometabolic risk in children. Wright et al. [32] perform hierarchical clustering and dimension reduction through treelet transformations to examine how dietary patterns are associated with periodontitis. Côté and Lamarche [33] provide an overview of current and future applications of machine learning to dietary research, focusing on the ways that complex interactions and diverse data can be integrated using these techniques.

Beyond these works, some authors have considered measurement error in machine learning for diet‐health problems. Russo and Bonassi [34] provide guidance to the application of neural networks in the context of nutritional epidemiology. With regard to the problem of measurement error, they discuss the use of technology to reduce errors that are present, emphasize that collecting high‐quality data is key to the successful application of neural networks, and indicate that expanding the size of data to increase statistical power may be useful in overcoming errors. We extend this advice to demonstrate that merely increasing the sample size will be insufficient to overcome the issues of measurement error. Morgenstern et al. [35] discuss how advances in machine learning may be able to be used to overcome challenges faced in nutritional epidemiology, with a particular emphasis on problems of measurement error. Their focus is on how data collection can be improved through the application of machine learning techniques, minimizing the impact of errors from the outset. Collecting high‐quality data that are free from errors is preferable to trying to correct for the effects of measurement error; however, most dietary intake data that are being collected or have previously been collected will be subject to measurement error. As a result, techniques to ensure valid analyses in the presence of errors remain important.

Neural networks are effectively a “black box algorithm,” in that it is difficult to credibly understand the rationale for generating a particular prediction [36]. As a result, it is critical to validate the specific underlying assumptions prior to the application of neural networks in any decision‐making context. In this work, we provide a quantitative consideration of the impacts of measurement error on neural network predictive models, informed by considerations relevant to dietary intake data. We demonstrate, through extensive simulations, possible concerns with the application of machine learning techniques in this context without careful consideration of error mechanisms. Our results suggest that, while there is substantial promise in the use of neural networks to capture the complex relationships within diet and health relationships, the same types of concerns that arise in traditional statistical modeling of these relationships are present when using neural networks and need to be carefully considered.

Specifically, we demonstrate how transformations of error‐prone data can improve predictive performance in dietary models, we illustrate the trade‐offs between an increase in sample size and an increase in the number of measurements taken for each individual, and we demonstrate concerns relating to overfitting that are accentuated in the presence of measurement error. Taken together, our results demonstrate that, without consideration of the underlying data structure, advanced computational techniques do not provide a notable benefit in predictive performance and come at the cost of lessened interpretability and increased computational time. We do not directly pursue specific corrections or modifications to machine learning techniques to overcome the impacts of measurement error on predictive modeling. However, our results suggest that, with care, there is reason to pursue the use of machine learning techniques for the analysis of dietary intake data. It is our hope that, with the impacts of errors and their possible causes outlined, future techniques may be developed to realize the promise of machine learning techniques applied to diet‐health relationships.

This article proceeds by introducing measurement error and neural networks, generally, before discussing how the former interacts with the latter in the context of dietary data in Section 2. We then illustrate, heuristically and theoretically, the concerns relating to using neural networks with error‐prone data in Section 3. In Section 4, the simulation experiments investigating the performance of neural networks subject to measurement error are detailed. The simulations form a case study based on data from the National Health and Nutrition Examination Survey (NHANES), rooting the analyses in real‐world scenarios. This connection is explored in more depth in Section 5, where the performance of neural networks and regression techniques is compared in an analysis of NHANES. Finally, Section 6 provides a discussion of the practical implications of our results.

## 2 Methodologies

We start by introducing the notation and measurement error models under consideration and the mathematical foundations of neural networks. In Section 2.1 we present the notation and underlying assumptions for the framework of error‐prone dietary intake data. We assume that outcomes are from a generalized regression framework, where some subset of the predictors are measured with error. Then, in Section 2.2, we introduce the statistical framework of predictive modeling, with a specific focus on the mathematical definition of neural networks.

### 2.1 Outcome and Measurement Error Models

Consider a response variable, Y, which is related to predictors {X,Z}. We take X to denote the predictors that are error‐prone and as such may not be observed accurately, while Z represents all other factors that are measured without error. We will assume that there are p error‐prone predictors and q error‐free predictors. In the simulations that follow, we will take a generalized regression framework, where we assume that

(1) $$E[Y|X,Z]=g^{-1}\left(\alpha_{0}+\alpha_{X}^{\prime }X+\alpha_{Z}^{\prime }Z\right)$$

where g(·) is a known link function and $\alpha ={\left(\alpha_{0},\alpha_{X}^{\prime },\alpha_{Z}^{\prime }\right)}^{\prime }$ is a vector of p+q+1 unknown parameters, with $\alpha_{X}\in {\mathbb{R}}^{p}$ and $\alpha_{Z}\in {\mathbb{R}}^{q}$. The conditional variance of Y given {X,Z} is denoted $\sigma_{Y}^{2}$. In this setting, $\sigma_{Y}^{2}$ may be taken to be $V(g^{-1}\left(\alpha_{0}+\alpha_{X}^{\prime }X+\alpha_{Z}^{\prime }Z\right))$ for a variance function, V, when appropriate.

In the measurement error literature, in place of X, we observe a surrogate measurement denoted $X^{*}$. We will often assume a specific functional form relating X and $X^{*}$, most commonly $X^{*}=X+V$, for a random error term V, with zero mean conditional on X and constant variance. This is the *classical additive* measurement error model. In estimation and inference problems, corrections for the effects of measurement error typically rely on auxiliary information, often in the form of *replicate* measurements. Instead of observing a single surrogate measurement, $X^{*}$, for each individual, several measurements are taken. This gives $\left\{X_{1}^{*},\ldots ,X_{m}^{*}\right\}$, which are typically assumed to be independent and identically distributed. Using these repeated measurements, information regarding the size and distribution of the errors can be determined.

In the case of dietary intake data, we take X to represent the usual intake of a particular dietary component, which is rarely directly measurable. Instead, we take the ℓth component of X (denoted $X_{\ell }$) to be an *unbiased* measurement of an individual's single‐day intake of a particular dietary component. Then, because true usual intake is the expected value of single day intake, any particular day's reported dietary intake is an error‐prone, surrogate measurement of usual intake, subject to error. Suppose that the reported intake for the ℓth dietary component on day j is denoted $X_{\ell ,j}^{*}$; then for each individual we define the usual intake $X_{\ell }=E[X_{\ell ,j}^{*}|Z]$. Take f(x) to be a Box‐Cox transformation [37]. We further assume that every recorded observation is given by

(2) $$X_{\ell ,j}^{*}=f^{-1}\left(\beta_{0,\ell }+\beta_{Z,\ell }^{\prime }Z+u_{\ell }+\epsilon_{\ell ,j}\right)$$

where $u_{\ell }$ captures the between‐individual variation for intake of the dietary component ℓ, and $\epsilon_{\ell ,j}$ captures the within‐person variation. $\beta_{\ell }={\left(\beta_{0,\ell },\beta_{Z,\ell }^{\prime }\right)}^{\prime }$ is a q+1 dimensional vector of unknown parameters.

In our numerical experiments, we assume that U is normally distributed with mean zero and variance $\sum_{u}$, denoting realizations as $u={\left(u_{1},\ldots ,u_{p}\right)}^{\prime }$. Further, for j=1,…,m, $\epsilon_{j}={\left(\epsilon_{1,j},\ldots ,\epsilon_{p,j}\right)}^{\prime }$ is assumed to be independent and identically distributed, according to a zero‐mean normal distribution with variance $\sum_{\epsilon }$. We note that the assumption that the ϵ terms are independent and identically distributed may be unrealistic in practice, with, for instance, the possibility of measurements that are closer in time being more highly correlated with one another. Still, we follow the convention in the nutrition literature, maintaining this assumption for expositional clarity [38]. We assume that U and $\epsilon_{\cdot }$ are independent of each other and of the outcome Y. We assume that these observations are made for a sample of size n, such that for each i=1,…,n the data consist of $\left\{Y_{i},X_{i}^{*},Z_{i}\right\}$, where $X_{i}^{*}=(X_{i,1}^{*},\ldots ,X_{i,p}^{*})$ and $X_{i,j}^{*}=(X_{i,1,j}^{*},\ldots ,X_{i,p,j}^{*})$ . In the case of dietary data, repeated measurements are captured by days of dietary measurements, and we use these expressions interchangeably.

When we take f(·) to be the identity and set $\beta_{Z,\ell }=0$, then we are left with $X_{i,\ell ,j}^{*}=\beta_{0,\ell }+u_{i,\ell }+\epsilon_{i,\ell ,j}$, where $u_{i,\ell }$ and $\epsilon_{i,\ell ,j}$ are both normally distributed and $\beta_{0,\ell }$ is an unknown constant. This is the classical additive model previously described. By allowing outcomes to depend on non‐linear transformations and on error‐free variates, the described measurement error model more accurately describes real‐world measurements of dietary intake data.

In this framework, the standard goal of inference and estimation centers on estimating α given the observed data. There are many techniques that exist that allow for the consistent estimation of regression parameters in this setting [15, 38]. In our investigation, we are concerned with α only insofar as it pertains to the prediction of Y. Our analytical goal is to develop a model that takes as input $\left\{X^{*},Z\right\}$ and outputs a prediction for Y. Given this notation, the aforementioned argument in favor of ignoring measurement error in predictive modeling can be expressed succinctly by noting that $X^{*}$ is an error‐free measurement of itself. Then, instead of modeling Y given {X,Z}, we can model Y given $\left\{X^{*},Z\right\}$ and use this model directly for prediction.

The need for flexible modeling in this setting becomes apparent in light of Equations (1) and (2). Even supposing that the link functions are known, the interaction of these models is complex, generally meaning that

$$E[Y|X^{*},Z]\neq g^{-1}({\tilde{\alpha }}_{0}+{\tilde{\alpha }}_{X}^{\prime }\tilde{h}(X^{*})+{\tilde{a}}_{Z}^{\prime }Z)$$

for a vector $\tilde{\alpha }$ and transformation $\tilde{h}(X^{*})$. An exception to this exists when g(·) is taken to be the identity. This gives a linear model for the conditional expectation of Y given {X,Z}, and in this case we can write that,

$$\begin{array}{ll} E[Y|X^{*},Z] & =E\left[E[Y|X^{*},X,Z]|X^{*},Z\right]\\ & =\alpha_{0}+\alpha_{X}^{\prime }E[X|X^{*},Z]+\alpha_{Z}^{\prime }Z \end{array}$$

This model is linear in $E[X|X^{*},Z]=E[X_{j}^{*}|Z]$. If f(·) is also the identity, then this will render the entire model linear, which means that $E[Y|X^{*},Z]$ takes the same form, up to the parameters, as E[Y|X,Z].

Even when linear models are assumed, a slight extension of Equation (1) is commonly applied where, instead of taking the outcome to be linear in X, some function is applied to the dietary components, transforming them. We call this function h, such that h(X) returns the relevant predictors of the outcome of interest that are related linearly to g(E[Y|X,Z]). This could include, for instance, products or ratios of multiple components. In this case,

(3) $$E[Y|X,Z]=g^{-1}\left(\alpha_{0}+\alpha_{h}^{\prime }h(X)+\alpha_{Z}^{\prime }Z\right)$$

where $h:{\mathbb{R}}^{p}\to {\mathbb{R}}^{k}$ and $\alpha_{h}$ is a k×1 vector. Using this model, even if h is known and both g(·) and f(·) are linear, it will not generally be the case that $E[Y|X^{*},Z]$ is linear in a transformed version of $X^{*}$.

### 2.2 Predictive Models and Neural Networks

The goal of the analysis is to construct a function of the observable data that produces estimates of the outcome. That is, we want to construct an estimator, $\hat{T}(X^{*},Z)$, that produces $\hat{y}=\hat{T}(X^{*},Z)$ as an estimate for Y. We are predominantly concerned with the accuracy of predictions generated by $\hat{T}$ as measured by a loss function of interest, say ${ℒ}_{\hat{T}}(Y,\hat{y})$. The loss function of interest will depend on the type of outcome that is observed and the specific context that the prediction model should be used in. In our work, we are predominantly concerned with the mean squared error (MSE). For predictions generated on a set of individuals, $i=1,\ldots ,n_{\text{test}}$, the MSE is given by

(4) $$ℒ(\hat{T})={ℒ}_{\hat{T}}(Y,\hat{y})=\frac{1}{n_{\text{test}}}\overset{n_{\text{test}}}{\underset{i=1}{\sum }}{\left[Y_{i}-\hat{T}(X_{i}^{*},Z_{i})\right]}^{2}$$

It is important to distinguish between the loss that is observed within the *training data* and the loss that is observed within the *testing data*. Training data refer to data that are used to estimate $\hat{T}$, while testing data are data that are withheld from the model fitting procedure used exclusively to test predictive performance. While it is generally preferable to have a small loss on both the training and testing data, in practice, performance on the testing data is of more value. If a model performs substantially better on training data than it does on testing data, we say that the model is *overfitting* the data. Testing data are meant to serve as more representative of the actual use case for the model, where predictions are generated for previously unseen data. The MSE computed on a validation sample is sometimes referred to as the *mean squared prediction error* (MSPE).

**FIGURE 1:**
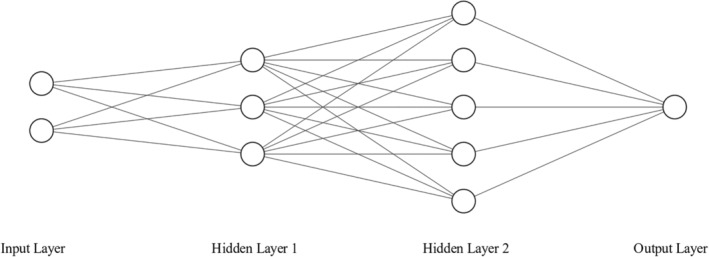
An example architecture for a fully connected neural network. Here the input layer has 2 nodes ($n^{\left[1\right]}=2$), the first hidden layer has 3 nodes ($n^{\left[2\right]}=3$), the second hidden layer has 5 nodes ($n^{\left[3\right]}=5$), and the output layer has a single node ($n^{\left[4\right]}=1$). With this structure, we are using 2 predictors to predict a univariate outcome.

Any method of model fitting can be applied in this setting. For instance, with a known link function, it may be natural to pursue prediction through generalized linear models or similar regression‐based techniques. However, the previously indicated complexity renders techniques that make fewer parametric assumptions particularly amenable to this setting. One such approach is the use of neural networks. Neural networks are well‐suited to the task as they are *universal approximators*, meaning that neural networks can approximate any relationship observed in data arbitrarily well, regardless of the complexity of the functional form generating the data [39, 40]. A neural network consists of *nodes* that are arranged into sequential *layers*. The first layer consists of one node for every input to the network. This input layer is connected to one or more *hidden layers*, which are connected in sequence to a final layer that has the same dimension as Y (in the case of a continuous outcome, this is a size of 1). There can be as many hidden layers, with as many nodes per layer, as is computationally feasible. We denote the size of the jth layer $n^{\left[j\right]}$. An example architecture is presented in Figure 1.

In a fully connected network, each node in a layer is connected via an edge to each node in the next layer. Each of these edges is assigned a particular numeric weight. The value at a particular node is computed by taking the weighted sum of all nodes at the previous layer, weighted by the edge weights connected to the current node, and then applying an *activation function* to the resulting sum. The activation function can be any scalar function, though, they are often selected to be nonlinear to introduce additional nonlinearity into the network. Once the value for a node is computed, it is pushed forward through the network, until the final outcome is produced.

Denote the value at the jth node in the ith layer as $\omega_{ij}$. Then $\omega_{1j}$ is given by the jth predictor input into the model, and for an L layer neural network, $\omega_{L\cdot }$ is the predicted output. Take $\varphi_{i}(\cdot )$ to be the activation function in the ith layer. Then for i∈{2,…,L}, $\omega_{ij}=\varphi_{i}\left(\sum_{\ell =1}^{n^{\left[i-1\right]}}\nu_{\ell ,j}^{\left[i-1\right]}\omega_{i-1,\ell }\right)=\varphi_{i}(a_{ij})$. In this expression, $\nu_{\ell ,j}^{\left[i-1\right]}$ is the weight for the connection between the ℓth node on layer (i−1), and the jth node on the ith layer. A feedforward, fully connected neural network's architecture is thus characterized by the number of layers (L), the sizes of the layers ($n^{\left[i\right]}$), and the activation functions used at each layer ($\varphi_{i}(\cdot )$).

The activation functions throughout the hidden layers are selected to introduce nonlinearity into the model. While it is conceptually possible to use any function for this purpose, some see more use in practice. For instance, the *ReLU* (φ(x)=max{0,x}), the *sigmoid* ($\varphi (x)={\left(1+\exp(-x)\right)}^{-1}$), and the *tanh* (φ(x)=tanh(x)) are all commonly used activation functions. In contrast to the hidden layers, the activation function for the output serves as a method for transforming the predicted values to the relevant scale or range of the output space. The activation function for the output layer can be thought of as analogous to the link function from a generalized regression framework. In fact, this similarity is more than aesthetic. If we take a two‐layer neural network, with $n^{\left[2\right]}=1$ as the output layer, and define $\varphi_{2}(x)=g^{-1}(x)$, then the predicted outcome has the same form as in Equation (1). While the estimation procedure for regression coefficients differs from the procedure for estimating neural network weights, this demonstrates that neural networks are a strict generalization of the introduced parametric regression framework.

The process of *fitting* a neural network is the process of estimating (or *learning*) the sets of weights, $\nu_{\cdot }^{\left[\cdot \right]}$, based on a training data set, and a specified architecture. Typically, neural networks are fit via stochastic gradient descent (SGD) with backpropagation [41]. This is an efficient technique for optimizing a loss function over the space of network weights. SGD is a gradient descent technique that replaces the computed gradient across all the data with an estimate of the gradient computed on a randomly selected sample from the data. This reduces the computational burden of updating throughout the optimization process, particularly when the gradients are extremely large. In practice, SGD replaces updates of the form $\nu^{\prime }=\nu +\zeta \nabla ℒ$ with

$$\nu^{\prime }=\nu +\frac{\zeta }{m}\overset{m}{\underset{i=1}{\sum }}\nabla {ℒ}_{i}$$

where m is the size of the batches used for the approximation. Backpropagation is an algorithm that efficiently computes the gradient of the loss function with respect to the weights of the network.

In general, the larger the network, both in terms of the number of layers and the number of nodes within each layer, the more capable the neural network is of learning complex relationships. However, larger networks require more computational resources and more data to fit and are more prone to overfitting than smaller networks [42]. As a result, selecting an architecture for a neural network involves searching for a sufficiently small neural network that accurately captures the present relationships. A drawback to using neural networks is the cost of training. Neural networks generally require a large amount of data to fit in a stable manner and can take an exceptionally long time, even on powerful hardware, to train until convergence (particularly when compared to, for instance, regression models with closed form solutions).

## 3 Considerations for Predictive Modeling With Measurement Error

In this section, we explore theoretically the application of neural networks to error‐prone data. In Section 3.1, we consider data transformations that can be applied to error‐prone variables to result in an approximately additive error structure. We demonstrate how, depending on the assumed measurement error model, these transformations can result in variance reduction when averaging error‐prone predictors. Section 3.2 explores how measurement error can increase the likelihood of a neural network to *overfit* data. We argue that the problem of overfitting is likely to be exacerbated when the input data are subject to error. Finally, Section 3.3 considers how trading off an increased sample size for an increased number of replicate measurements may result in an overall improvement to the predictive model's performance. These results suggest that, contrary to existing advice in the literature, there are diminishing returns to an increased sample size when data are error‐prone, if sufficient numbers of replicate measurements have not been taken.

### 3.1 Error Variance Reduction and Transformations to Additivity

When considering data measured with error, intuitively, the smaller the error variance, the better a predictive model will perform. The rationale is that there is less noise distorting the relationship between the outcome and the informative components of the predictors. While it is not possible to directly reduce the error variance without changing the instrument being used for measurement, it is generally possible to simulate the effects of a variance reduction by averaging repeated measurements of the predictors. Suppose that we directly observe $X_{\ell ,j}^{*}$ as in Equation (2), for j=1,…,k. The measurement error in this observation is captured through the term $\epsilon_{\ell ,j}$ with variance $\sigma^{2}$. Take f(x)=x and consider

$$\begin{array}{ll} {\bar{X^{*}}}_{\ell }^{\left(+\right)} & =\frac{1}{k}\overset{k}{\underset{j=1}{\sum }}X_{\ell ,j}^{*}=\beta_{0,\ell }+\beta_{Z,\ell }^{\prime }Z+u_{\ell }+\frac{1}{k}\overset{k}{\underset{j=1}{\sum }}\epsilon_{\ell ,j}\\ & =\beta_{0,\ell }+\beta_{Z,\ell }^{\prime }Z+u_{\ell }+{\bar{\epsilon }}_{\ell } \end{array}$$

The error term in ${\bar{X_{\ell }^{*}}}^{\left(+\right)}$ is ${\bar{\epsilon }}_{\ell }$, which has variance $\sigma^{2}/k$. Thus, when there is an additive structure for the measurement error, averaging provides a reduction in variance by a factor equal to the number of days that are averaged. In this sense, averaging is equivalent to using an improved instrument with a reduced variance. As k→∞, the error term ${\bar{\epsilon }}_{\ell }$ vanishes.

Suppose instead that f(x)=log(x), so that the daily observed values are subject to an exponential transformation. Then

$$\begin{array}{ll} {\bar{X^{*}}}_{\ell } & =\frac{1}{k}\overset{k}{\underset{j=1}{\sum }}X_{\ell ,j}^{*}=\frac{1}{k}\overset{k}{\underset{j=1}{\sum }}\exp\left\{\beta_{0,\ell }+\beta_{Z,\ell }^{\prime }Z+u_{\ell }+\epsilon_{\ell ,j}\right\}\\ & =\exp\left\{\beta_{0,\ell }+\beta_{Z,\ell }^{\prime }Z+u_{\ell }\right\}\left(\frac{1}{k}\overset{k}{\underset{j=1}{\sum }}\exp\left\{\epsilon_{\ell ,j}\right\}\right) \end{array}$$

Averaging here reduces the error variance; however, this reduction is smaller than in the linear case. If we improved the instrument we were using to give the same variance as the average in this case, this corresponds to an error term with variance

$$\sigma_{\text{Reduced}}^{2}=\log\left[\frac{1}{2}\left(\sqrt{\frac{k-4e^{\sigma^{2}}+4e^{2\sigma^{2}}}{k}}\right)+1\right]$$

So long as k≥2, then we have that $\sigma^{2}/k\leq \sigma_{\text{Reduced}}^{2}\leq \sigma^{2}$. Thus, the resulting error variance is reduced by averaging; however, the reduction in variance is smaller than the reduction for an additive model.

Suppose that instead of taking ${\bar{X^{*}}}_{\ell }$ under the exponential transform, we use a log transform, and then retransform the variables exponentially. That is, consider the quantity

$$\begin{array}{ll} {\tilde{X^{*}}}_{\ell } & =\exp\left(\frac{1}{k}\overset{k}{\underset{j=1}{\sum }}\log(X_{\ell ,j}^{*})\right)=\exp\left({\bar{X^{*}}}_{\ell }^{\left(+\right)}\right)\\ & =\exp\left\{\beta_{0,\ell }+\beta_{Z,\ell }^{\prime }Z+u_{\ell }\right\}\exp\left\{{\bar{\epsilon }}_{\ell }\right\} \end{array}$$

From the above argument, we know that the error variance of this term is less than averaging on the untransformed scale. In general, if the transformation f is sufficiently smooth, $f({\bar{X^{*}}}_{\ell }^{\left(+\right)})$ will have smaller variance in the measurement error term than ${\bar{X^{*}}}_{\ell }$.

Lemma 1 *Subject to regularity conditions of* f, *then taking* $\epsilon_{i}\overset{iid}{∼}N(0,\sigma^{2})$ *independently of* Ω *for* i=1,…,k, *we have that*

$$\mathrm{var}\left[\;f(\Omega +\bar{\epsilon })|\Omega \right]\leq \mathrm{var}\left[\left.\frac{1}{k}\overset{k}{\underset{i=1}{\sum }}f(\Omega +\epsilon_{i})\right|\Omega \right]$$

Lemma 1 tells us that if the transformations we are considering are particularly well‐behaved (for instance, exponential functions or polynomials), then it is the case that averaging over an additive model before reapplying the transformation will result in a quantity with less variance than averaging the observed quantities. The exact transformation will dictate the degree to which the variances are altered. Eckert et al. [43] discuss techniques for transforming error‐prone variables to a scale on which they are additive, providing techniques for obtaining this variance reduction. This result does not necessarily mean that the transformed average predictor will be a better predictor of the truth in terms of MSE, but rather that the transformed average predictor will be equivalent to an error‐prone instrument with less overall variance compared to the average transformed predictor. If $\epsilon_{j,\ell }$ contains predictive information beyond that contained in Z and $u_{\ell }$, then reducing the measurement error variance will not generally improve predictive performance.

We assumed that the predictors were defined as

$$X_{\ell }=E[X_{\ell ,j}^{*}|Z,u_{\ell }]=E[f^{-1}(\beta_{0,\ell }+\beta_{Z,\ell }^{\prime }Z+u_{\ell }+\epsilon_{\ell ,j})|Z,u_{\ell }]$$

This means that, in our setting, we are assuming that the errors, $\epsilon_{\ell ,j}$, are informative. This is in contrast to assuming

$$\begin{array}{ll} X_{\ell } & =f^{-1}\left\{E[f(X_{\ell ,j}^{*})|Z,u_{\ell }]\right\}\\ & =f^{-1}\left\{E[\beta_{0,\ell }+\beta_{Z,\ell }^{\prime }Z+u_{\ell }+\epsilon_{\ell ,j}|Z]\right\}\\ & =f^{-1}\left\{\beta_{0,\ell }+\beta_{Z,\ell }^{\prime }Z+u_{\ell }\right\} \end{array}$$

Under this assumption, the errors are non‐informative, and analysts should consider finding transformations that render the error‐prone predictors additive on a linear scale. These predictors can be averaged before being transformed to the scale used for prediction. This process will effectively reduce error variance, potentially improving the predictive performance of the models.

### 3.2 Measurement Error and Neural Network Overfitting

Overfitting refers to the problem of a predictive model performing substantially better on a training set compared with a validation set. For a particular estimation procedure resulting in the prediction model $\hat{T}$, take the loss on the training set to be $\tilde{ℒ}(\hat{T})=\tilde{ℒ}$ and the loss on the testing set to be $ℒ(\hat{T})=ℒ$. Generally, we expect that $\tilde{ℒ}<ℒ$, but in a well‐calibrated model the difference should be minimal. As the size of the validation sample increases, Equation (4) indicates that ℒ will converge almost surely to $E[ℒ]=E\left[{\left(Y-\hat{T}(X^{*},Z)\right)}^{2}\right]$. The degree of overfitting in a model can then be assessed by the degree to which E[ℒ] exceeds $\tilde{ℒ}$.

The best predictor, in terms of MSE, for Y, observing $\left(X^{*},Z\right)$ is $\hat{T}(X^{*},Z)=E[Y|X^{*},Z]$, resulting in a minimal E[ℒ] of $\mathrm{var}(Y|X^{*},Z)$. When the true variates, X, are available, $E[ℒ]\geq \sigma_{Y}^{2}$. Consider taking $X_{\ell ,j}^{*}=\beta_{0,\ell }+\beta_{Z,\ell }^{\prime }Z+u_{\ell }+\epsilon_{\ell ,j}$ and $X_{\ell }=\beta_{0,\ell }+\beta_{Z,\ell }^{\prime }Z+u_{\ell }$. Here $X_{\ell ,j}^{*}=X_{\ell }+\epsilon_{\ell ,j}$. The conditional variance of Y given $\left\{X^{*},Z\right\}$ is

$$\begin{array}{ll} \mathrm{var}(Y|X^{*},Z) & =\mathrm{var}\left(\left.g^{-1}(\alpha_{0}+\alpha_{X}^{\prime }(X^{*}-\epsilon )+\alpha_{Z}^{\prime }Z)\right|X^{*},Z\right)\\ & \;+\sigma_{Y}^{2}>0+\sigma_{Y}^{2} \end{array}$$

where the inequality follows since the first term has a residual random component that is not fully specified by $X^{*}$ and Z. This means that the lower bound on the validation loss when subject to measurement error, in this setting, will exceed the lower bound for the validation loss in the error‐free setting.

Despite the fact that the theoretical limits of performance on testing data favor the error‐free context, this says nothing of the capacity for neural networks to fit the training data. Previous work has shown that, for many architectures of neural networks, adding noise to the input data does not lead to an increased training loss [44]. In other words, even when there is no true pattern to learn, neural networks show a capacity to encode the training data exactly. In our context this means that, for a given neural network, we may not expect $\tilde{ℒ}$ to differ substantially between the cases when X is available or when $X^{*}$ are available.

Combining these two results leads to the conclusion that, in the presence of measurement error, neural networks have a greater capacity to overfit the training data when compared to training on error‐free data. This is a result that is confirmed in our simulation experiments (Section 4.1). Overfitting can be addressed through sufficient validation, regularization techniques, and carefully selected model architectures. However, the increased capacity to overfit presents challenges to the application of neural networks in dietary intake studies. Practitioners must be careful to ensure that techniques to prevent overfitting are applied and that training performance does not lend undue confidence to the out‐of‐sample performance of the model. It is also worth emphasizing that overfitting does not mean that the performance of the neural network will be poor on out‐of‐sample data or that it will suffer when compared to alternative techniques. Instead, overfitting renders the training error to be an incorrectly calibrated assessment of the true model performance, and this problem is exacerbated by the presence of measurement error.

### 3.3 Sample Size and Replicate Counts for Predictive Power

In order to increase the size of data that are used to train a predictive model, it is possible to increase either the sample size, n, or the replicate counts, k. Typically in dietary assessment research it will be the case that n≫k. It is worth considering how these two quantities impact the performance of a predictive model. To do so, consider a simple case that is analytically tractable. Suppose that $Y=g^{-1}(X)+e$, with a univariate $X∼N(\mu_{X},\sigma_{X}^{2})$, and $e∼N(0,\sigma_{Y}^{2})$ independently of all other random variables. Further, suppose that $X^{*}=X+V$ for $V∼N(0,\sigma_{V}^{2})$ independently of all other random variables. If $g^{-1}$ is sufficiently smooth in an interval around E[X] such that $g^{-1}(X)\approx g^{-1}(E[X])+\frac{d}{dX}g^{-1}(E[X])(X-E[X])$ from a first order Taylor series, then

$$\mathrm{var}(g^{-1}(X)|X^{*})\approx {\left[\frac{d}{dX}g^{-1}(E[X])\right]}^{2}\mathrm{var}(X|X^{*})$$

The best predictor for Y given $X^{*}$ has a lower bounded loss of $\mathrm{var}(Y|X^{*})=\mathrm{var}(g^{-1}(X)|X^{*})+\sigma_{Y}^{2}$. By assumption, $X=X^{*}-V$, and as a result $\mathrm{var}(X|X^{*})=\mathrm{var}(X^{*}-V|X^{*})=\mathrm{var}(V|X^{*})$. In this simplified setting we conclude that $X^{*}∼N(\mu_{X},\sigma_{X}^{2}+\sigma_{V}^{2})$ and that

$$\mathrm{var}(V|X^{*})=\frac{\sigma_{V}^{2}\sigma_{X}^{2}}{\sigma_{V}^{2}+\sigma_{X}^{2}}$$

Instead of $X^{*}$ being a single observation, we take $X^{*}=\frac{1}{k}\sum_{j=1}^{k}X_{j}^{*}$, where each $X_{j}^{*}=X+V_{j}$ with $V_{j}$ independent and identically distributed according to $N(0,\sigma_{V}^{2})$. Then $X^{*}$ will be normally distributed with mean $\mu_{X}$ and variance $\frac{1}{k}\sigma_{V}^{2}+\sigma_{X}^{2}$, and

$$\mathrm{var}(V|X^{*})=\frac{\sigma_{V}^{2}\sigma_{X}^{2}}{\sigma_{V}^{2}+k\sigma_{X}^{2}}$$

The difference from using repeated measurements comes in the form of a variance reduction, on the order of 1/k. Put differently, as more replicates of $X_{j}^{*}$ are observed, the lower bound for the predictive loss continues to improve. As k grows to ∞, $\mathrm{var}(Y|X^{*})$ converges to var(Y|X), and prediction with the error‐prone variates can be done as well as prediction with the error‐free variates. This suggests that, in the event that the functional relationship $E[Y|X^{*}]$ is exactly known, it remains beneficial to increase the number of replicate observations for each individual in order to improve out‐of‐sample predictive performance.

Normally, the functional relationship defining $E[Y|X^{*}]$ will not be explicitly known and will need to be estimated based on observed data. Suppose that $E[Y|X^{*}]=h(X^{*})$, and that this relationship is consistently estimated from a sample of size n. Then

$$\begin{array}{ll} E\left[{\left(Y-{\hat{h}}_{n}(X^{*})\right)}^{2}|X^{*}\right] & =E\left[{\left(Y-h(X^{*})\right)}^{2}|X^{*}\right]\\ & \;+E\left[{\left(h(X^{*})-{\hat{h}}_{n}(X^{*})\right)}^{2}|X^{*}\right] \end{array}$$

The first term is simply $\mathrm{var}(Y|X^{*})$, exactly as in the previous analysis. The second term is the estimation MSE. While the structure of neural networks renders the task of finding bounds on convergence rates challenging, current theoretical findings in the deep learning literature suggest that, in at least some settings, deep learning can achieve near parametric optimal rates in terms of the MSE [45, 46, 47]. That is to say, the second term, in the best‐case scenario, is of order $n^{-1+\delta }$, for an arbitrarily small δ>0.

Combining these two results, the residual prediction MSE, in an idealized scenario, will be $O\left(k^{-1}+n^{-1+\delta }\right)$. That is,

$$E\left[{\left(Y-{\hat{h}}_{n}(X^{*})\right)}^{2}|X^{*}\right]=\sigma_{Y}^{2}+\frac{C_{1}}{k}+\frac{C_{2}}{n^{1-\delta }}$$

for constants $C_{1}$ and $C_{2}$ that depend on $\sigma_{V}^{2}$ and $\sigma_{X}^{2}$. Depending on the specific breakdown of $\sigma_{V}^{2}$ and $\sigma_{X}^{2}$, the relative magnitudes of $C_{1}$ and $C_{2}$ will differ. In dietary research, we have a comparatively small k, often with k≤2, and a comparatively large n, often with n in the thousands. Correspondingly, even when $C_{2}\gg C_{1}$, the scaling factors are likely to be such that an increase in k will have a more sizable decrease in the overall error than an increase in n. In fact, when these results hold, it may be the case that halving n and doubling k such that n×k remains constant may provide a more efficient allocation of resources in terms of overall predictive performance. This is a result that we explore in more depth, for relative variances that are reasonable in the context of nutrition data, in simulation experiments (Section 4.2). The general pattern we observe, as indicated by this heuristic theory, is that when a large enough sample is taken for adequate model performance, it is advantageous for model performance to increase k, even if that means reducing n.

Two important considerations stem from these results. First, it is critical to understand the convergence behavior of neural networks in the presence of measurement error. It is not sufficient to take a large n to overcome the impacts of measurement error on prediction. Even in theoretically infinite samples, the models that are fit to data that are subject to measurement error will perform worse than models fit to data that are free from error. Depending on the exact setting, this degradation in performance can be substantial. Instead, convergence to the optimal model requires the simultaneous increase of both n and k. As a result, when studies are to be designed, it remains important to sufficiently budget for repeated measurements, even when prediction is the goal. Generally, dietary intake data have k≤2, owing to cost and data quality constraints. These results suggest that, even for predictive modeling, study designs should consider achieving higher levels of replication whenever possible. Second, when a model is fit with a set number of replicates, k, future predictions implicitly assume the same level of replication. If a predictive model is fit using data from 2 days of replicates, it will not be advantageous to record more than 2 days of information for out of sample prediction. Additionally, prediction will rely on being able to collect 2 days of replicated information out‐of‐sample as well. This requirement may be overcome through distributional assumptions and transformations; however, in practice it is prudent to design the study with these considerations in mind.

## 4 Measurement Error in Neural Networks

We now consider numerical experiments to investigate the impacts of measurement error on the predictive performance of neural networks applied to data that are subject to error. We begin by illustrating how the potential benefits of using neural networks for prediction may be undermined in the presence of measurement error. We then give evidence that increasing the number of available replicate measurements can lead to improved predictive performance. Next, we show how, if there is sufficiently large measurement error, neural networks do not outperform incorrectly specified linear regression models, even in highly nonlinear settings. Finally, we demonstrate that the impacts of measurement error in settings for binary prediction appear similar. Taken together, these results suggest that, while there is a strong motivation for finding valid methods of applying neural networks to the analysis of dietary intake data, analysts must proceed with caution in the application of these techniques. Unless otherwise specified, the simulations consider neural networks with 5 layers, with each layer consisting of 50 nodes. A dropout proportion of 0.001 is used, with batch normalization applied and ReLU activation functions. The batch sizes are taken to be 1000, and the Adam optimizer is used with a learning rate of 0.1 and a weight decay of 0.0001. The scheduler reduces the learning rate by a factor of 10 every 300 epochs. The networks are trained for a maximum of 1500 epochs and checked for early stopping every 50 epochs. In the training data, 15% is held out for validation, and an unmodified MSE is used for the loss function.

The structure for the neural network was arrived at by considering the impact of the hyperparameters in a variety of experiments performed prior to these simulations. These simplified versions of the problem were used to assess the size of the network, as well as the network's performance across a range of parameter values for the optimizer. We used the RandomSearchCV method from the Skorch package to perform cross‐validation on the parameter values in these simplified scenarios.

### 4.1 Transformations and Overfitting

The first scenario assumes there are two error‐prone variates, $X_{1}$ and $X_{2}$, and no error‐free variates. We take $h(X)=\frac{X_{1}}{X_{2}}$ so that

$$Y=\alpha_{0}+\alpha_{1}\frac{X_{1}}{X_{2}}+e$$

where $e∼N(0,\sigma_{Y}^{2})$, independent of all other variates. We assume that we observe two days' worth of replicates, giving for each individual $\left\{X_{11}^{*},X_{12}^{*},X_{21}^{*},X_{22}^{*}\right\}$, where we generate $X_{\ell ,j}^{*}=\beta_{0,\ell }+u_{\ell }+\epsilon_{\ell ,j}$, with ${\left[\begin{array}{ll} u_{1} & u_{2} \end{array}\right]}^{\prime }∼N(0,\sum_{u})$ and ${\left[\begin{array}{ll} \epsilon_{1,j} & \epsilon_{2,j} \end{array}\right]}^{\prime }∼N(0,\sum_{\epsilon })$ independent of all variates for each j, and we take

$$X_{\ell }=E\left\{{\left[\lambda X_{\ell ,j}^{*}+1\right]}^{1/\lambda }\right\}.$$

We take $\sigma_{Y}^{2}=1$, $\beta_{01}=36$, $\beta_{02}=27.5$, $\alpha_{0}=98.5$, $\alpha_{1}=4.0$, $\sum_{U}=\left[\begin{array}{ll} 20 & 15.5\\ 15.5 & 25.5 \end{array}\right]$, $\sum_{\epsilon }=\left[\begin{array}{ll} 38 & 20.5\\ 20.5 & 34.5 \end{array}\right]$, and λ=0.35. We consider replicating measurements for 2,4,6,8, and 10 days, with a sample size of 12 000 (giving 12 000 observations per day).

The analysis proceeds, comparing linear regression models with neural networks. For each method we consider three different data transformations to prepare the data:

1. averaging the error‐prone measurements across each day of observation (averaging);
2. treating each observed, error‐prone measurement as a separate predictor (concatenation); or
3. performing a Box‐Cox transformation of the error‐prone measurements and then averaging these results (transformed averaging).

**FIGURE 2:**
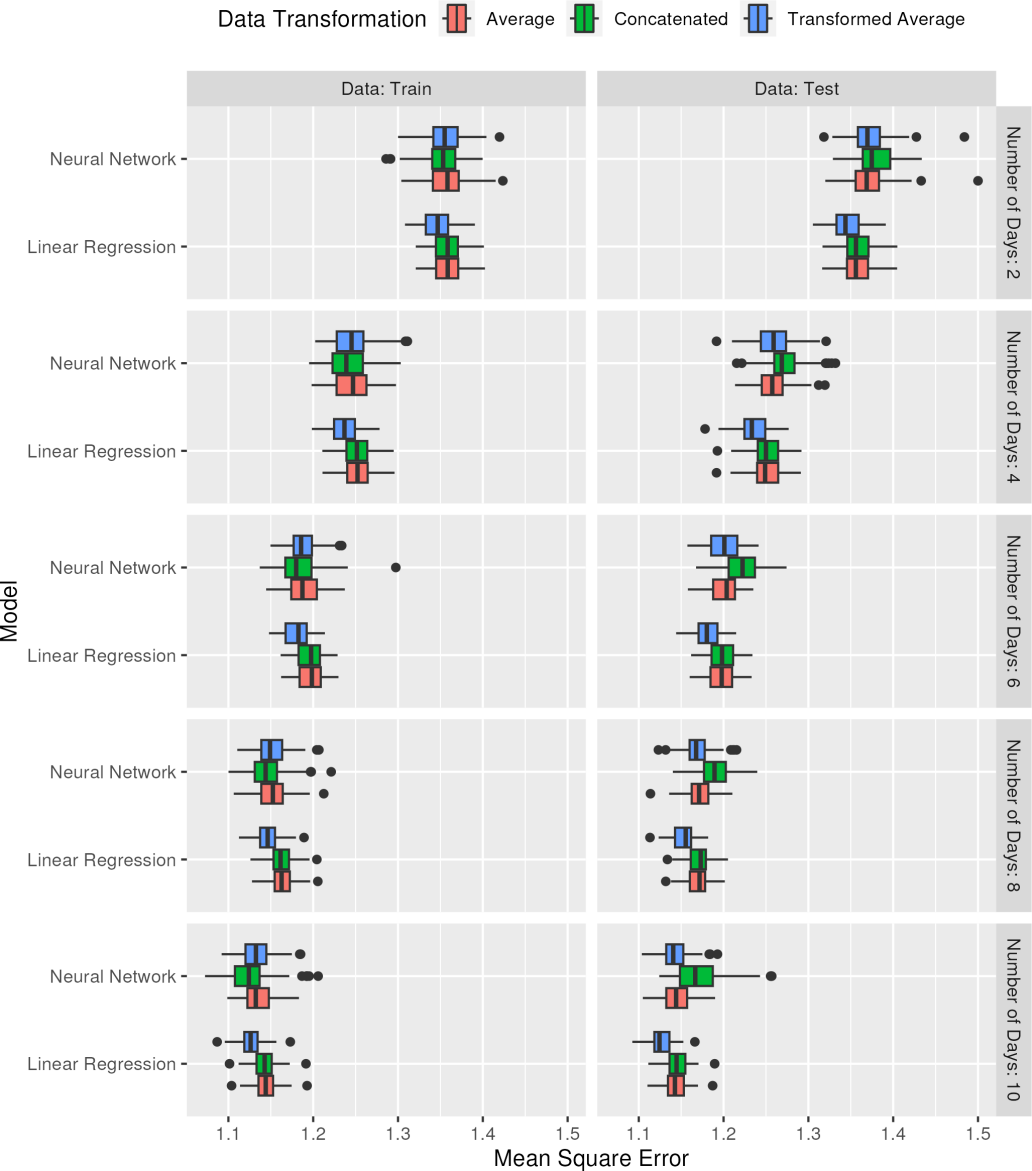
MSE for both neural networks and linear regression models predicting an outcome based on the ratio of predictors, where each predictor is subject to additive measurement error, and the sample size is 12 000. Results are shown for both the training and testing data, and over an increasing number of days, where each individual has measurements taken for the predictors each day.

Note, the linear regression model is incorrectly specified since $X_{1}^{*}$ and $X_{2}^{*}$ are passed as linear predictors, rather than as a ratio. The neural network also takes each of the predictors linearly. Figure 2 contains the MSE for each of the various scenarios, plotted by number of days, model type, and data preparation. The results are included for both the training data and the testing data.

The results demonstrate the expected pattern that, as more data are used to predict outcomes, the overall MSE decreases in both the training and testing sets. The neural networks tended to perform better than the corresponding linear regression model in the training data and performed worse than the linear regression models on the testing data, though these differences tend to be fairly minor. The neural network seems to overfit the training data, no matter the number of days, when each of the predictors is provided independently (concatenation), as evidenced by the fact that the concatenated results are the strongest in the training set and the weakest in the testing set. This is in line with the theoretical observations, where in the training data the neural network is able to use noise as a signal, despite the fact that this does not generalize to the testing data. With the linear regression, using the transformed average consistently outperformed all other techniques in terms of MSE. Within the neural networks, both averaging approaches performed similarly, and these tended to outperform the concatenated results on the test set.

### 4.2 Sample Size and Replicate Counts

**FIGURE 3:**
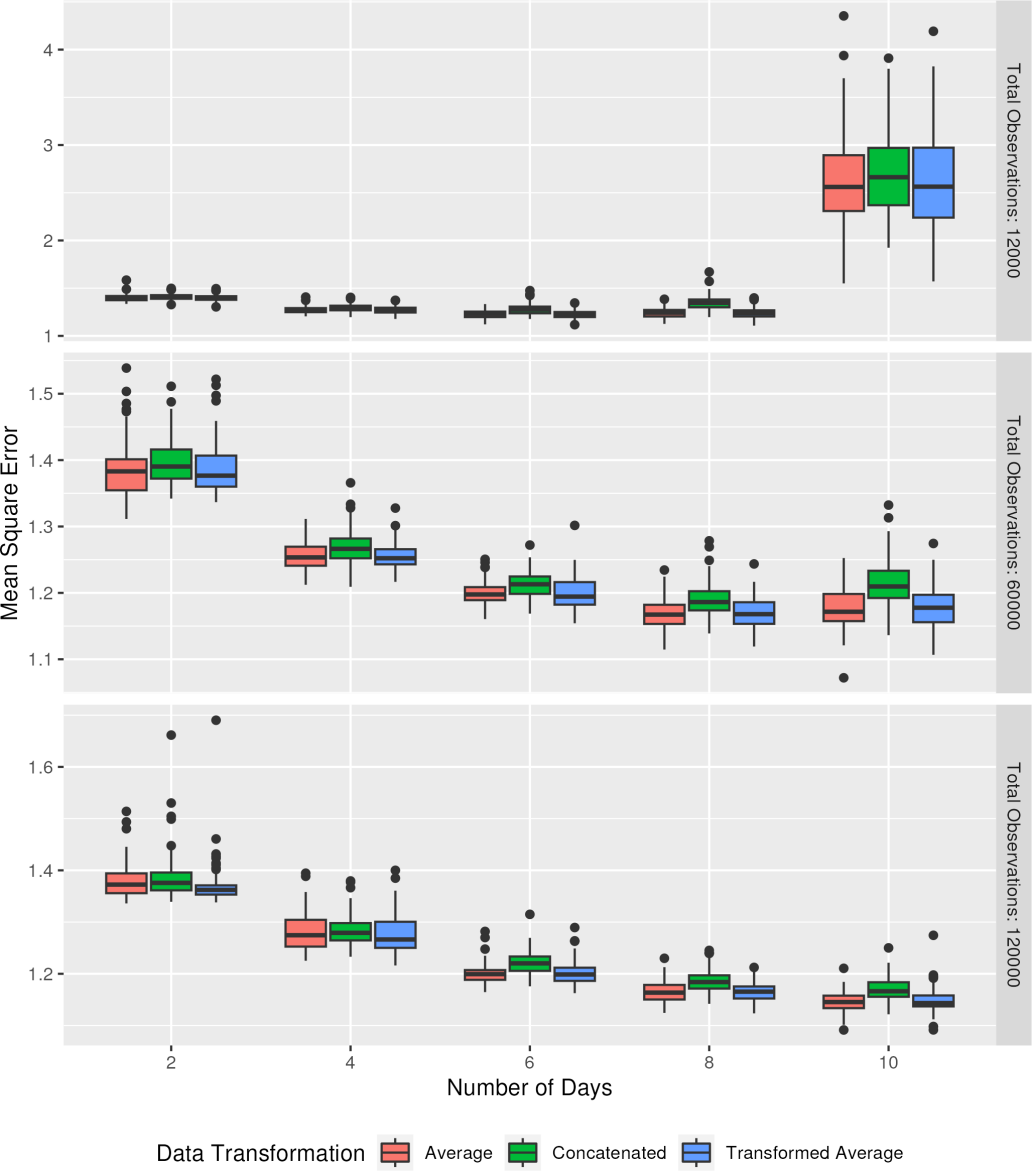
MSE for neural networks predicting an outcome based on the ratio of predictors, where each predictor is subject to additive measurement error. The total number of observations is fixed at 12 000, 60 000, or 120 000 and divided evenly between the differing number of days. Results are shown for the testing data.

In the second simulation experiment, we consider the same scenario as in the previous subsection, with λ=0.5 rather than λ=0.35, increasing the skewness of the simulated data. In these simulations, we vary the number of replicated days that we are averaging over, keeping the total number of measurements constant across the sample. This allows us to investigate the trade‐off between the benefits of a larger sample with the benefits of smaller error variances. In particular, we consider using 2, 4, 6, 8, or 10 days' worth of replicates. We fix the total number of measurements to be either 12 000 (with samples sizes 6000,3000,2000,1500, or 1200), 60 000 (with samples sizes 30000,15000,10000,7500, or 6000), or 120 000 (with sample sizes 60000,30000,20000,15000, or 12000). In Figure 3 the MSE for the neural network, with each of the data transformations outlined in simulation 1, is shown.

The results indicate that, all else equal, it is preferable to increase the number of days of replicates of the error‐prone variables, even if that means sacrificing the total sample size. This pattern is evident when using 60 000 or 120 000 total observations, but not when using 12 000, suggesting that there is a minimum sample size required for the neural network to provide stable prediction. This suggests that it is worth considering the minimal sample sizes required to achieve stable results for specific scenarios prior to deploying these methodologies. While not shown, regression analyses were run, with results similar to those presented in the first simulation. Additionally, the same overfitting of the concatenated results occurs. Comparing 60 000 and 120 000 total observations, the results are fairly similar, indicating that there may be a point of diminishing returns for this model.

### 4.3 Prediction of Outcomes With Non‐Linearity

In the third simulation experiment, we consider a scenario that depends on nonlinear functions of the truth,

$$\begin{array}{ll} Y & =\alpha_{0}+\alpha_{1}X_{1}+\alpha_{2}X_{2}+\alpha_{3}\frac{X_{1}}{X_{2}}+\alpha_{4}\log(X_{1})\\ & \;+\alpha_{5}\log(X_{2})+\alpha_{6}\sqrt{X_{1}X_{2}}+Z_{3}+e \end{array}$$

where e∼N(0,1) independently of all other factors. We take Z to be multivariate normal, with mean 0 and covariance

$$\sum_{Z}=\left[\begin{array}{llll} 1.28 & 0.21 & -0.07 & 0.32\\ 0.21 & 1.98 & 1.28 & 0.34\\ -\;0.07 & 1.28 & 1.91 & 1.22\\ 0.32 & 0.34 & 1.22 & 1.20 \end{array}\right].$$

We generate $X={\left[\begin{array}{ll} X_{1} & X_{2} \end{array}\right]}^{\prime }$ to be drawn from the conditional distribution

$$X|Z∼N\left(\beta {\left[\begin{array}{lllll} 1 & Z_{1} & Z_{2} & Z_{3} & Z_{4} \end{array}\right]}^{\prime },\left[\begin{array}{ll} 20 & 15.5\\ 15.5 & 25.5 \end{array}\right]\right).$$

We take the error‐prone observations to be $X_{\ell ,j}^{*}=X_{\ell }+\epsilon_{\ell ,j}$, for j=1,2, and ${\left[\begin{array}{ll} \epsilon_{1,j} & \epsilon_{2,j} \end{array}\right]}^{\prime }∼N(0,\sum_{\epsilon })$ independently of all other variables, where $\sum_{\epsilon }=\left[\begin{array}{ll} 38 & 20.5\\ 20.5 & 34.5 \end{array}\right]$. We take a sample size of n=40000 for the training and testing samples, and repeat the simulations 100 times.

**FIGURE 4:**
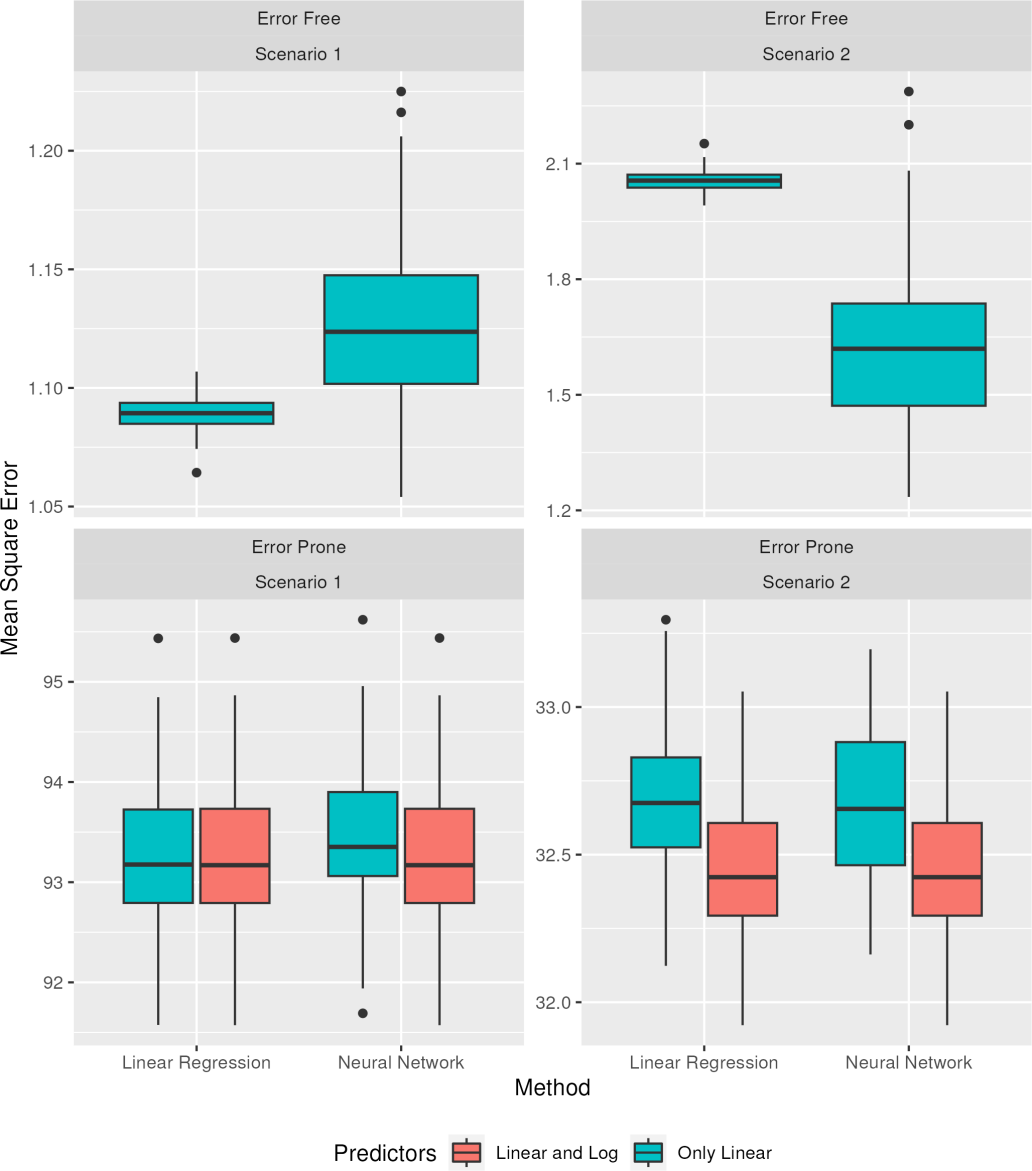
MSE for linear regression and neural networks predicting an outcome based on a nonlinear function of predictors, for both the error‐free and error‐prone settings. Two separate scenarios are considered, each using two days of replicates. Results are shown for the testing data.

Two scenarios are formed, varying α and β. Scenario 1 takes $\alpha ={\left[\begin{array}{lllllll} 350 & 2 & -1 & 3 & 2 & 1 & -4 \end{array}\right]}^{\prime }$, and $\beta =\left[\begin{array}{lllll} 100 & 2 & 0 & -1 & 0.5\\ 100 & 0 & 2 & 1 & -0.5 \end{array}\right]$. Scenario 2 takes $\alpha ={\left[\begin{array}{lllllll} 350 & 1 & -1 & 50 & 25 & 25 & -1 \end{array}\right]}^{\prime }$, and $\beta =\left[\begin{array}{lllll} 50 & 2 & 0 & -1 & 0.5\\ 50 & 0 & 2 & 1 & -0.5 \end{array}\right]$. For each scenario, we consider both error‐free and error‐prone models. For the error‐free models, we consider only the true predictors as linear terms. For the error‐prone models, we consider both the models that include the error‐prone predictors as linear terms and ones that also include the log‐transformed predictors. These predictors are averaged over the two days of observations. The MSE for each technique across both scenarios on the test data is displayed in Figure 4.

Across both error‐prone scenarios, the linear regression and neural network perform comparably when using the same predictors. For both methods, inclusion of the log terms improves the MSE. This difference is more pronounced in scenario 2, where the log terms have a larger impact on the outcome. These results are best understood when compared to the error‐free context. Both the linear regression and neural network are able to attain near the theoretical lower bound on MSE in scenario 1, when using only the linear predictors. For scenario 2, the neural network substantially outperforms the linear regression, when considering only linear predictors. This improvement is degraded, however, with the introduction of measurement error.

### 4.4 Binary Prediction

In the fourth simulation experiment, we consider predicting a binary outcome that is dependent on highly nonlinear relationships with the variates. Specifically, we take

$$\begin{array}{ll} & P(Y=1|X)\\ & \;=\text{expit}\left(-1+\frac{1}{30}X_{2}[I(X_{1}\geq 100)-I(X_{3}\leq 30)]+\frac{1}{4}\log(X_{3})\right) \end{array}$$

where $\text{expit}(x)={\left(1+\exp(-x)\right)}^{-1}$. We generate $X={\left[\begin{array}{lll} X_{1} & X_{2} & X_{3} \end{array}\right]}^{\prime }$ to be drawn from a multivariate normal with mean vector ${\left[\begin{array}{lll} 100 & 50 & 30 \end{array}\right]}^{\prime }$ and covariance

$$\sum =\left[\begin{array}{lll} 25 & 20 & 5\\ 20 & 20 & 8\\ 5 & 8 & 21 \end{array}\right]$$

We take two days worth of replicate observations, giving $X_{\ell ,j}^{*}=X_{\ell }+\epsilon_{\ell ,j}$ for j=1,2, where

$$\left[\begin{array}{l} \epsilon_{1j}\\ \epsilon_{2j} \end{array}\right]∼N\left(0,\left[\begin{array}{lll} 16 & 32 & 16\\ 32 & 80 & 36\\ 16 & 36 & 53 \end{array}\right]\right)$$

**FIGURE 5:**
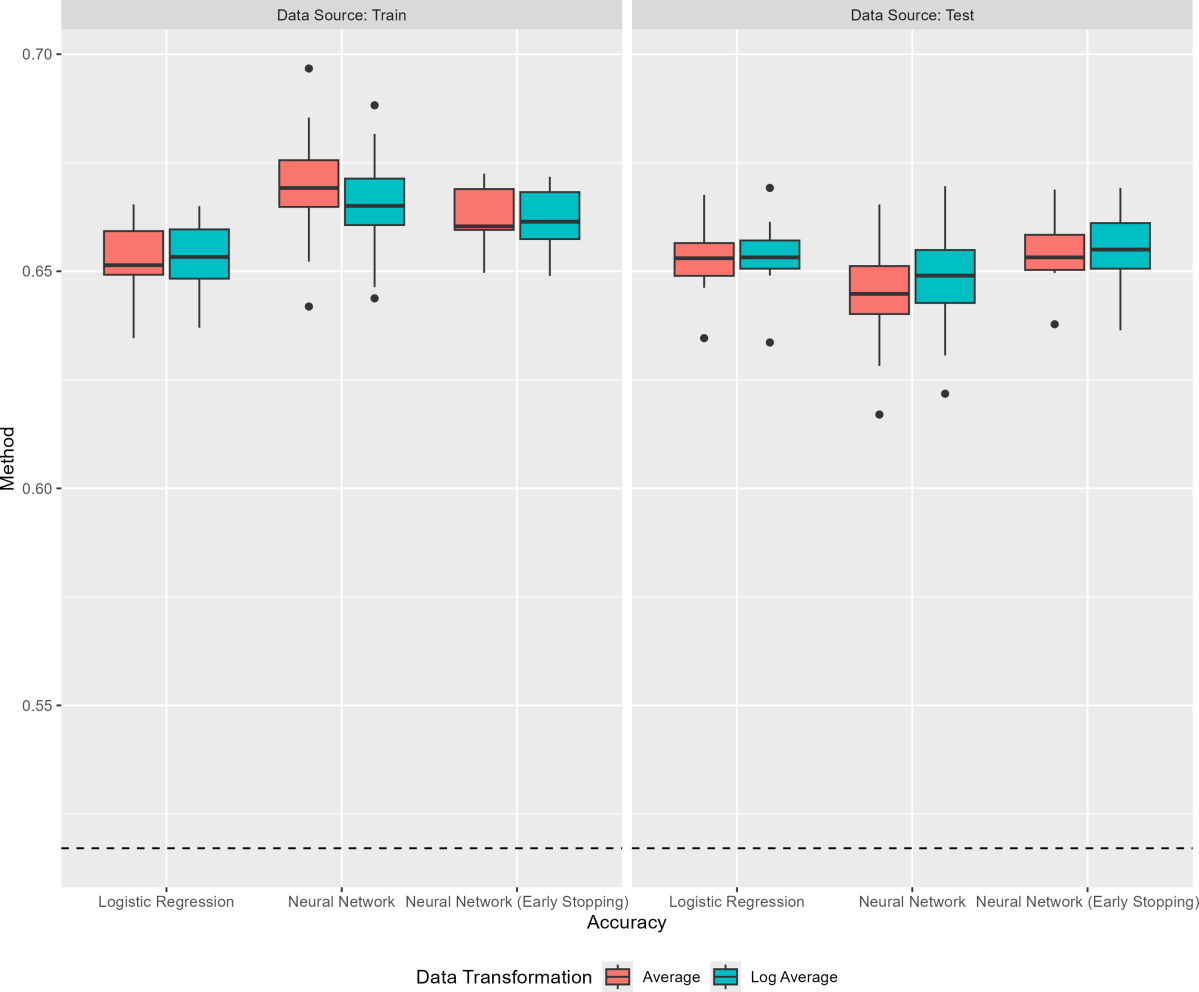
Accuracy for logistic regression and neural networks predicting a binary outcome based on a nonlinear function of predictors. Two separate neural networks are considering, one with early stopping after every 50 epochs and batch sizes of 1000 and one with early stopping after every 10 epochs and batch sizes of 250 (labeled “Early Stopping”). The dotted line indicates the median level of accuracy for a constant model. Results are shown for both the training and testing data.

We take a sample size of n=10000 for the training and testing samples and repeat the simulation 100 times. For each scenario, we consider two separate neural network training procedures as well as a logistic regression. The first neural network uses a batch size of 1000 and checks for early stopping after every 50 epochs. The second uses a batch size of 250 and checks for early stopping after every 10 epochs. For the neural networks and the logistic regression, we consider the performance of the methods when they receive either the average of each days' intake or else, where they receive the average of days' one and two and the log‐transformed average of day 3. As a result, all models remain misspecified. The prediction accuracy for each method and run is summarized in Figure 5.

The performance of these methods, in terms of overall accuracy, is relatively similar. The neural network with early stopping outperforms the other methods when it receives the log‐transformed predictors, with the incorrectly specified logistic regression following closely behind. All methods outperform the constant model. The relative performance of the neural networks is improved in this setting compared to the settings with continuous outcomes; however, it should be noted that this comparison remains against a misspecified regression model rather than against the truth. This is a distinct advantage of the semiparametric nature of neural networks. In this context, in order to achieve similar performance using the logistic regression, it would require more deliberate model selection and variable transformations, a step that is somewhat alleviated using the neural networks. This benefit is expected to be conferred whenever highly complex, nonlinear relationships (such as those simulated) are present.

## 5 Data Analysis

To demonstrate the caution required when applying deep learning predictive models to error‐prone data, we conduct an analysis of a subset of NHANES data from the 2005–2010 waves [48]. These data are collected using 24HRs, with processes that have adapted and evolved over time based on recommendations and emerging data needs [49]. Generally, two 24HRs are attempted to be collected for each individual; however, some respondents have missing second measurements.

The considered subset is inspired by the analysis conducted by Zhang et al. [50]. These authors investigated the association between usual sodium and potassium intakes and blood pressure. Adults aged 20 and older with reliable information on their 24HRs (where reliability is determined by the data collectors) were included in the data (N=15702). We removed individuals who had a missing value for the measured outcome (N=618). Additionally, individuals who were pregnant, reported being on a low sodium diet, or reported taking anti‐hypertensive medication were excluded from the analytic sample (N=4516 were excluded). The self‐reported intakes of sodium and potassium (measured in mg) were included as the error‐prone predictors, with up to two measurements taken per individual. The average diastolic blood pressure measurements were taken as the outcome. The predictors that are assumed to be measured without appreciable error are the individual's reported gender, age, cardiovascular disease status, educational attainment (beyond twelve years or less than twelve years total), reported ethnicity, reported smoking status, reported diabetes status, and an indicator for reported kidney disease status. Individuals with any missing, error‐free predictors were excluded from the analysis (N=14 further individuals were excluded). In total, there were N=10554 individuals in the final data.

Once collected, the data were artificially split into a training set (N=8442) and a testing set (N=2112). These data were partitioned to include equivalent proportions of individuals who had a single repeated measure of each sodium and potassium (N=1076 and N=270 in the training and testing sets, respectively). Note: any individual who had a second measure for sodium had one for potassium, and vice versa. Once split, the training data were used to fit and select linear regression models and neural networks. These fitted models were evaluated on the testing set in terms of the MSE.

The structure and hyperparameters of the neural networks were selected via 10‐fold cross‐validation. Three different approaches, one without early stopping, (one with early stopping checks every 20 epochs, and one with early stopping checks every 60 epochs), were used when fitting the neural networks. Each network was also fit with either the average of the individual days or with the multiple measurements concatenated. Prior to fitting, the data were standardized (to have 0 mean and unit variance). These analyses were performed in Python, using the PyTorch library [51].

Three separate linear regression models were fit to the data. The first two took the data as they had been modified for the neural network (one using the concatenation, one using the average of the two days) and fit a simple linear model with linear terms and no interactions. The third linear regression model followed a more rigorous modeling procedure, as would likely be applied in practice. First, a multivariate power transform was fit to the bivariate predictors. These transformed responses were averaged for all individuals, and a predictor containing the number of observations (either 1 or 2) was included. Then, starting from a model that contained all identifiable three‐way interactions, a backwards search procedure occurs to select the best subset model based on 10‐fold cross‐validation. This procedure is implemented with the R packages leaps [52] and caret [53]. Running this procedure selected a model that contained 44 terms in addition to the intercept, where, in addition to the predictors themselves, two‐ and three‐way interactions are considered a single term. The sensitivity of these results was considered by taking the smallest model that was within 0.01 of the 44‐term model in terms of cross‐validated root mean square error (the square root of MSE). This led us to consider a 19‐term model as well.

The resulting MSE for each of the models on both the training and testing data are shown in Table 1. First, we note that the performance of the linear regression model selected through the backwards selection procedure, which kept only 19 terms, was nearly identical to that of the backwards selection procedure, which was optimal and kept 44 terms, demonstrating a lack of sensitivity. Moreover, we can see that both of the naive regression models performed worse (on the training and testing data) than the more rigorously justified regression model, as is anticipated from the theory.

**TABLE 1:** The training and testing MSE for the neural networks and linear regression models fit to the NHANES data, based on an 80%/20% train/test split. Bolded values indicate the most performant techniques for the training and testing sets, respectively.

Model	Training MSE	Testing MSE
Neural network (Averaged, no early stopping)	128.26	142.12
Neural network (Concatenated, no early stopping)	126.59	142.88
Neural network (Averaged, early stopping–20 Epochs)	129.90	143.08
Neural network (Concatenated, early stopping–20 Epochs)	129.83	143.69
Neural network (Averaged, early stopping–60 Epochs)	130.06	142.69
Neural network (Concatenated, early stopping–60 Epochs)	128.75	143.41
Linear regression (Averaged)	138.71	146.87
Linear regression (Concatenated)	138.70	146.79
Linear regression (Backwards selection, Size = 44)	132.26	142.05
Linear regression (Backwards selection, Size = 19)	132.46	142.05

Comparing the neural networks and regression models, the neural networks (using either averaged or concatenated data) performed slightly worse than the regression models on the testing set, despite improved training performance. A stark difference exists for the networks trained on the concatenated data. These outperformed all other techniques on the training data; however, they were less effective than either of the selected regression or the neural network with averaged data on the testing set. This is indicative of the same overfitting behavior demonstrated in the numerical studies. It is important to note that this overfitting behavior was observed even when taking explicit steps to avoid its impact.

While performance is comparable between the neural networks and linear regression models, it is important to consider the costs of applying machine learning in this setting. The neural networks require substantially more computational time and resources to fit and produce results that are less interpretable than the linear regression. Moreover, the fitted neural network models do not lend themselves to inferential procedures, meaning that prediction is the only utility of the model. It may well be the case that, in this setting, primary interest is not in prediction itself, but rather in inference regarding underlying associations, medical interventions, or so forth. In these settings, purely predictive models, such as neural networks, are at a disadvantage compared to alternative statistical approaches. Given that the performance is not improved in this setting and may be slightly degraded, caution in the application of these techniques is warranted.

## 6 Discussion

Dietary intake data are complex and error‐prone. There is an ongoing effort to leverage the powerful techniques emerging from machine learning research to analyze diet‐health relationships, and this research has shown promising results. However, concerns specifically relevant to dietary intake data, particularly as they relate to measurement error, have not been previously addressed in the statistical or computing literature. The most prominent modern machine learning techniques, including artificial neural networks, are primarily predictive models. The literature on the impact of measurement error on prediction models is sparse, owing largely to the fact that in many settings it can simply be ignored. However, the errors that are common in dietary intake data are often large enough to dramatically reduce the performance of prediction models, resulting in a strong motivation to consider avenues for improvement. This is salient when these predictive models are intended to be used to make decisions or predict health outcomes of individuals and populations using dietary intake data.

We investigate the impact of classical measurement error on the performance of neural networks in settings that are analogous to those confronted in nutrition epidemiology research. Even though this preliminary work does not attempt to address the complex measurement error structures comprising systematic bias as well as noise that are thought to apply to self‐report dietary data, we demonstrate that even well‐behaved measurement error is cause for concern. We show that, as expected, error reduces model performance for neural networks, and this reduction can be sizable. We demonstrate how, when these errors are sufficiently large, the signal becomes nearly undetectable, rendering misspecified linear regression models equally effective at out‐of‐sample prediction in many scenarios. In highly complex, nonlinear scenarios, the semiparametric nature of neural networks allows for modestly improved prediction over the misspecified regression models. We illustrate theoretically and via empirical simulations that neural networks are prone to overfitting data that are subject to measurement error, and avoiding this requires particular care during model development. We note that, while the patterns of underperformance are present across most of the considered scenarios, we have not exhaustively investigated all settings where prediction with error‐prone data may be used. Our theoretical results indicate why this may be a challenging problem, however, the performance of different predictive models should be investigated with the nuances of specific settings present. We suggest that prediction in these settings may be better calibrated by considering multiple prediction techniques that may be differently impacted by measurement errors as a form of sensitivity analysis. Future work that minimizes the impacts of measurement error in these settings, or more efficiently uses auxiliary data, may produce more promising predictive results.

Beyond the shortcomings of neural networks, however, we also saw the promise of improved performance at detecting complex relationships. The ability of networks to detect highly nonlinear relationships, without the need to explicitly codify these relationships, seems the most promising result in our simulations. These results can be further improved with care towards data collection and model fitting. To this end, we showed that models can benefit dramatically from increasing replicate data. In particular, we showed that for predictive models, there are likely to be diminishing returns in increasing the sample size beyond a particular point; however, increasing the number of measurements per individual can continue to improve the model performance. When designing a study that will be used to fit a predictive model, it is important to consider the number of replicates that can be measured, even if this results in a decreased overall sample size. In practice, increasing the number of observations per individual results in reduced data quality, which may undermine the observed results. Our results stand in contrast to standard practice in the nutrition surveillance literature, where it is often suggested that two recalls suffice for data collection. Instead, these results lend further credibility to authors who have suggested that more than two replicates are preferred, even accounting for the degradation of data quality [54]. Further research that considers how to make use of data with distributional shift, or that further takes into account alternative dietary assessment mechanisms (such as food frequency questionnaires), may help to resolve this theory‐practice gap. Using FFQs alongside 24HRs presents alternative concerns relating to the systematic biases present in FFQs. Still, this combined approach has been investigated with some promise for traditional techniques [55]. We illustrated how, depending on the underlying structure of the data, transformations to additivity may serve to improve the variance reduction attained through averaging procedures. These transformations have been well‐studied in the measurement error literature, and they may serve an important role in making the most of the available replicates.

In light of our results, we will also stress the importance of model specification for predictive modeling. Results reliably improved when the specified models were closer to the truth or when hyperparameters were selected through cross‐validation. These techniques should be applied, regardless of the underlying modeling technique. Our results indicate the importance of tailoring solutions to the specific prediction problem that is being considered. For instance, it may be possible to more efficiently leverage the repeated days measurements in a neural network, rather than relying on simple averaging. Future work may consider modified network structures or modified loss functions in order to directly leverage these replicates more efficiently. Our results do not suggest that deep learning is entirely inappropriate for prediction in these settings but rather that further work is needed to realize the full promise of these techniques.

Overall, this work serves to stress the important point that, while neural networks are powerful semiparametric prediction models, they are subject to the same concerns present in traditional statistical methodologies. Not only that, but these techniques tend to be computationally expensive and nearly uninterpretable. When they are able to deliver superior predictive performance, these tradeoffs are likely worthwhile. However, our work demonstrates that, without care to the structure of the data that are available, it is unlikely that theoretically defensible results can be reliably obtained in dietary research by applying machine learning techniques directly. Our work serves to demonstrate some preliminary areas of focus for the valid application of machine learning techniques to data that are prone to measurement error, and leaves open many questions regarding the best approach to leveraging the power of neural networks for nutritional epidemiology research. Further simulation and theoretical work is required to expand these results and in particular, to make concrete recommendations for overcoming the outlined issues. In the interim, however, care is required when deploying black box prediction models in these settings.

## Conflicts of Interest

The authors declare no conflicts of interest.

## Supporting information


Data S1

## Appendix A

### Conditions on f: Proof of Lemma 1

Suppose that there is a convergent Taylor series representation of f(x) such that

$$f(x)=\overset{\infty }{\underset{r=1}{\sum }}\frac{f^{\left(r\right)}(a)}{r!}{\left(x-a\right)}^{r}$$

We wish to compare the conditional variance of $f(\Omega +\bar{\epsilon })$ with $\bar{f(\Omega +\epsilon )}$, given Ω. The evident choice is to take a=Ω, so that

$$\begin{array}{ll} & f(\Omega +\bar{\epsilon })=\overset{\infty }{\underset{r=1}{\sum }}\frac{f^{\left(r\right)}(\Omega )}{r!}{\bar{\epsilon }}^{r}\\ & \bar{f(\Omega +\epsilon )}=\frac{1}{k}\overset{k}{\underset{j=1}{\sum }}\overset{\infty }{\underset{r=1}{\sum }}\frac{f^{\left(r\right)}(\Omega )}{r!}\epsilon_{j}^{r}\\ & \mathrm{var}\left[\;f(\Omega +\bar{\epsilon })|\Omega \right]\\ & \;=\overset{\infty }{\underset{r=1}{\sum }}\overset{\infty }{\underset{r^{\prime }=1}{\sum }}\text{cov}\left(\left.\frac{f^{\left(r\right)}(\Omega )}{r!}{\bar{\epsilon }}^{r},\frac{f^{\left(r^{\prime }\right)}(\Omega )}{r^{\prime }!}{\bar{\epsilon }}^{r^{\prime }}\right|\Omega \right)\\ & \;=\overset{\infty }{\underset{r=1}{\sum }}\overset{\infty }{\underset{r^{\prime }=1}{\sum }}\frac{f^{\left(r\right)}(\Omega )f^{\left(r^{\prime }\right)}(\Omega )}{r!(r^{\prime })!}\text{cov}\left({\bar{\epsilon }}^{r},{\bar{\epsilon }}^{r^{\prime }}\right)\\ & \;=\overset{\infty }{\underset{r=1}{\sum }}\overset{\infty }{\underset{r^{\prime }=1}{\sum }}\frac{f^{\left(r\right)}(\Omega )f^{\left(r^{\prime }\right)}(\Omega )}{r!(r^{\prime })!}\left(E[{\bar{\epsilon }}^{r+r^{\prime }}]-E[{\bar{\epsilon }}^{r}]E[{\bar{\epsilon }}^{r^{\prime }}]\right)\\ & \;=\overset{\infty }{\underset{r=1}{\sum }}\overset{\infty }{\underset{r^{\prime }=1}{\sum }}\frac{f^{\left(r\right)}(\Omega )f^{\left(r^{\prime }\right)}(\Omega )}{r!(r^{\prime })!}{\left(\frac{\sigma }{\sqrt{k}}\right)}^{r+r^{\prime }}\\ & \;\times \left(I(r+r^{\prime }\;\text{even})(r+r^{\prime })!!-I(r,r^{\prime }\;\text{even})r!!(r^{\prime })!!\right)\\ & \;=\overset{\infty }{\underset{r=1}{\sum }}\overset{\infty }{\underset{r^{\prime }=1}{\sum }}C(\Omega ,r,r^{\prime })\frac{\sigma^{r+r^{\prime }}}{k^{(r+r^{\prime })/2}}\\ & \mathrm{var}\left[\;\bar{f(\Omega +\epsilon )}|\Omega \right]\\ & \;=\frac{1}{k^{2}}\overset{k}{\underset{j=1}{\sum }}\overset{\infty }{\underset{r=1}{\sum }}\overset{k}{\underset{j^{\prime }=1}{\sum }}\overset{\infty }{\underset{r^{\prime }=1}{\sum }}\text{cov}\left(\left.\frac{f^{\left(r\right)}(\Omega )}{r!}\epsilon_{j}^{r},\frac{f^{\left(r^{\prime }\right)}(\Omega )}{(r^{\prime })!}\epsilon_{j^{\prime }}^{r^{\prime }}\right|\Omega \right)\\ & \;=\frac{1}{k^{2}}\overset{k}{\underset{j=1}{\sum }}\overset{\infty }{\underset{r=1}{\sum }}\overset{\infty }{\underset{r^{\prime }=1}{\sum }}\text{cov}\left(\left.\frac{f^{\left(r\right)}(\Omega )}{r!}\epsilon_{j}^{r},\frac{f^{\left(r^{\prime }\right)}(\Omega )}{(r^{\prime })!}\epsilon_{j}^{r^{\prime }}\right|\Omega \right)\\ & \;=\frac{1}{k}\overset{\infty }{\underset{r=1}{\sum }}\overset{\infty }{\underset{r^{\prime }=1}{\sum }}\text{cov}\left(\left.\frac{f^{\left(r\right)}(\Omega )}{r!}\epsilon^{r},\frac{f^{\left(r^{\prime }\right)}(\Omega )}{(r^{\prime })!}\epsilon^{r^{\prime }}\right|\Omega \right)\\ & \;=\overset{\infty }{\underset{r=1}{\sum }}\overset{\infty }{\underset{r^{\prime }=1}{\sum }}\frac{f^{\left(r\right)}(\Omega )f^{\left(r^{\prime }\right)}(\Omega )}{r!(r^{\prime })!}\frac{\sigma^{r+r^{\prime }}}{k}\\ & \;\times \left(I(r+r^{\prime }\;\text{even})(r+r^{\prime })!!-I(r,r^{\prime }\;\text{even})r!!(r^{\prime })!!\right)\\ & \;=\overset{\infty }{\underset{r=1}{\sum }}\overset{\infty }{\underset{r^{\prime }=1}{\sum }}C(\Omega ,r,r^{\prime })\frac{\sigma^{r+r^{\prime }}}{k} \end{array}$$

Consider that, for k≥2 and k>1, $k^{k}\geq k$, and so $\frac{1}{k^{k}}\leq \frac{1}{k}$. Noting that $r+r^{\prime }\geq 2$ for all $r,r^{\prime }$ in the summation, we get that

$$\begin{array}{ll} C(\Omega ,r,r\prime )\frac{\sigma^{r+r\prime }}{k^{(r+r\prime )/2}} & \leq C(\Omega ,r,r\prime )\frac{\sigma^{r+r\prime }}{k}\;\Rightarrow \;\mathrm{var}\left[\;f(\Omega +\bar{\epsilon })|\Omega \right]\\ & \leq \mathrm{var}\left[\;\bar{f(\Omega +\epsilon )}|\Omega \right] \end{array}$$
